# Cross-sectional study of the association of body composition and physical fitness with bone status in children and adolescents from 11 to 16 years old

**DOI:** 10.1186/1471-2431-13-117

**Published:** 2013-08-09

**Authors:** Anderson Marques de Moraes, Ezequiel Moreira Gonçalves, Vinicius Justino de Oliveira Barbeta, Gil Guerra-Júnior

**Affiliations:** 1 Department of Pediatrics, Growth and Body Composition Laboratory, Center for Investigation in Pediatrics (CIPED), Faculty of Medical Sciences (FCM), University of Campinas (UNICAMP), Campinas, SP 13083-887, Brazil

**Keywords:** Ultrasonography, Phalangeal, Fat mass, Physical activity, Body composition

## Abstract

**Background:**

The aim of the study was to verify the association between body composition and physical fitness with bone status in children and adolescents.

**Methods:**

A cross-sectional study was conducted with 300 healthy students (148 boys, 152 girls). Weight, height, fat and fat-free mass, and percentage of body fat (%BF) were evaluated, as were physical fitness (abdominal exercise, flexibility, and horizontal jump tests) and maximum oxygen consumption. Bone parameters (amplitude-dependent speed of sound; AD-SoS) and the Ultrasound Bone Profile Index (UBPI) were evaluated using DBM Sonic BP ultrasonography.

**Results:**

In the study group, girls had higher bone parameter values than boys. A univariate analysis assessed in a stepwise multiple regression model was conducted. It showed that for boys, the %BF and height were significant independent variables for AD-SoS and UBPI, but the horizontal jump test only for AD-SoS (adjusted r^2^ = 0.274; p < 0.001), and pubertal maturation only for UBPI (adjusted r^2^ = 0.295; p < 0.001). For girls, age and %BF were identified as significant independent variables for AD-SoS and UBPI (adjusted r^2^ = 0.093; p < 0.001) but height only for AD-SoS (adjusted r^2^ = 0.408; p < 0.001).

**Conclusions:**

Variables related to growth (age, height, and pubertal maturation) are independent positive predictors for the bone parameters in both boys and girls. %BF is an independent negative predictor. For boys, the horizontal jump test was an independent positive predictor for AD-SoS, indicating that physical fitness related to the neuromotor system can influence the amount of bone present.

## Background

Physical activity is a key factor for improving physical fitness related to health, and has been indicated as a major determinant of bone mass throughout life [1]. Accordingly, several studies have investigated its contribution to the accumulation of bone mass during childhood and adolescence [2-5]. The influence of physical fitness on bone density during adulthood is a way to understand the process of bone maturation and to identify factors that may contribute to more effective interventions, helping to create strategies to prevent bone-related diseases [6].

Several methods have been used to measure bone mass, including dual-energy X-ray absorptiometry (DXA) and peripheral quantitative computed tomography. However, in recent years, quantitative ultrasonography (QUS) of the phalanges has been used for indirect evaluation of bone tissue. The rationale is based on the results of a variety of experiments suggesting that US parameters provide information not only about the quantity but also about bone architecture and elasticity [7]. QUS has some practical advantages over other methods that use X-rays and photons (i.e., low cost, safety, freedom from ionizing radiation, and practicality), making it suitable for use in children and adolescents [8-10].

The aim of the present study was to verify the associations between body composition and physical fitness using the bone status in children and adolescents according to their sex.

## Methods

This cross-sectional study was conducted on 624 students from the Bradesco Foundation School (Campinas, Brazil) who were invited to participate. The sample was selected intentionally. The exclusion criteria were the presence of physical disability (permanent or temporary) that precluded conducting the evaluations (*n* = 2), use of drugs that might interfere with body composition or bone mass (*n* = 0), no consent to participate from the parents or students (*n* = 22), and no execution of any test or absence on any evaluation day (*n* = 300). The final sample consisted of 300 students: 148 boys (49.4%) and 152 girls (50.6%) aged 11–16 years. Chronological ages were established by calculating decimal ages, with reference to the birth date and survey date, using decimal intervals between 0.50 and 0.49 [11]. The Committee of Ethics in Research of the Faculty of Medical Sciences, University of Campinas approved the study. The school board and parents gave written informed consent.

### Anthropometry and body composition

Weight (kg) and height (m) were measured using standardized techniques [12]. Body mass index (BMI) was calculated as the ratio between weight (kg) and height (m^2^).

Triceps and medial calf skinfolds were measured according to the American College of Sports Medicine recommendations [13] using a specific skinfold caliper with 0.1-mm precision. The equations proposed by Slaughter et al. [14] for children and adolescents 8–18 years of age were used to calculate the percentage of body fat (%BF). Based on these data, fat mass was calculated by %BF × weight. The fat-free mass was calculated by subtracting the fat mass from the weight.

Pubertal maturation was performed by self-assessmen, using specific figures for breast stage (B1–5) for girls and male genitalia (G1–5) for boys according to the criteria of Marshall and Tanner [15,16]. The stages were prepubertal (stage 1), intrapubertal (stages 2 and 3), and pubertal (stages 4 and 5).

### Bone status

The third generation of DBM Sonic BP equipment (IGEA, Carpi, Italy) was used for determining the bone parameters: amplitude-dependent speed of sound (AD-SoS) and Ultrasound Bone Profile Index (UBPI). The equipment is fitted with a probe that attaches two transducers (transmitter and receiver). The probe is positioned at the distal metaphysis of each of the last four proximal phalanges (II–V) of the nondominant hand. The transducer transmitter emits a sound wave of 1.25 MHz, and the transducer receiver picks up the signal and assesses the speed of propagation of sound through the phalange [10]. The quantitative (AD-SoS) and qualitative (UBPI) parameters result from this assessment. The AD-SoS is obtained automatically and represents the average of speed measurements of the ultrasound (m/s) transmitted that tracked trabecular bone tissue on the four proximal phalanges. This parameter depends on the amplitude of the electrical signal, obtained after US has covered three types of bone in the phalanges (endosteal, trabecular, cortical). The UBPI is a combination of three US parameters calculated by signal analysis of the US signal. Fast-wave amplitude is the amplitude of the first US pulse that reaches the receiving probe once the US pulse has propagated throughout the phalange. Bone transmission time is the time needed for the US wave to propagate through the bone tissue alone. The signal dynamic—the sharpness of the first two US pulses that reach the receiving probe—is calculated as the second derivative of the amplitude by time. The software generates values between 0 and 1. Values that are closer to 1 indicate better bone quality [9,10].

The use of this method is interesting because the assessed site (distal metaphysis of proximal phalanges) has high metabolic activity during all stages of life and has great similarity to the microstructures of the lumbar spine [9]. The equipment for the technique has the advantages of being portable, noninvasive, and without radiation exposure. It is also easy to manipulate and has a low internal error that varies between 0.23% and 0.57% [8].

One evaluator (E.M.G.) performed all of the US measurements. *In vivo* short-term precision was assessed based on the root mean square of the coefficient of variation (RMS-CV) for 80 measurements made in 10 healthy young persons (six boys, four girls) measured four times each. It was calculated according to Bonnick et al. [17]. The RMS-CV values were 0.55% for AD-SoS and 5.72% for UBPI.

### Physical fitness

To determine physical fitness, a 1-min crunch abdominal exercise (abdominal strength), a sit-and-reach exercise (flexibility), and horizontal jump tests (power of the lower limbs) were performed according to the standardizations described by the American Alliance for Health, Physical Education, Recreation and Dance [18,19]. The shuttle run test proposed by Léger et al. [20] was performed to evaluate cardiorespiratory fitness (VO_2peak_). A team of experienced previously trained evaluators applied the tests. To avoid random errors, only one evaluator was responsible for the information obtained from each test.

#### Statistical analysis

The statistical analyses were performed using SPSS version 16.0 software (SPSS, Chicago, IL, USA). The normality of the data was verified using the Kolmogorov Smirnov test. When normal distribution was not observed, the transformation of Blom was completed to achieve homogeneity and normality of variables. Multivariate analysis of covariance was used to verify the differences in dependent variables between sex and pubertal development. Age was used as a covariate. The Bonferroni post-hoc test was used when necessary. Multivariate normality and homogeneity of variances and covariances were evaluated. Pearson’s correlation coefficient was used to verify correlations between the QUS parameter and anthropometric variables, body composition, and physical fitness. Stepwise multivariate linear regression analyses were performed to determine the possible effects of a group of independent variables (i.e., age, weight, height, BMI, fat mass, fat-free mass, %BF, pubertal stage, horizontal jumping ability, flexibility, abdominal test, and VO_2peak_) on the dependent variables (i.e., AD-SoS and UBPI). The results were considered statistically significant at p < 0.05.

## Results

### General characteristics

Anthropometric characteristics, body composition, physical fitness, and QUS parameters of the study group—total and separated by sex and pubertal maturation—are shown in Table 1. Significant differences (p < 0.01) were found between sex and AD-SoS, UBPI, and flexibility values, and they were greater in girls. The results by pubertal stage showed significant differences in weight, height, fat-free mass, AD-SoS, and UBPI (p < 0.01). There was an interaction between sex and pubertal maturation in the variables BMI, %BF, fat mass, UBPI, abdominal test, and horizontal jump test.

**Table 1:** Body composition, quantitative ultrasonography parameters, and physical fitness regarding the sex and pubertal stage of 300 students

	**Sex**		**Pubertal stages**		**Total**
**Variables**	**Girls**	**Boys**	**Pre/intra**	**Pubertal**	
	**(n = 104)**	**(n = 94)**	**(n = 124)**	**(n = 176)**	**(n = 300)**
**Weight (Kg)**	47.6 ± 1,3	51.1 ± 1.3	46.6 ± 1.1	52.0 ± 0.9^b^	49.8 ± 0.6
**Height (m)**	1.53 ± 0,006	1.56 ± 0.006	1.52 ± 0.007	1.58 ± 0.006^b^	1.55 ± 0.004
**BMI (Kg/m**^**2**^**)**	21.1 ± 0,3	20.6 ± 0.3	20.0 ± 0.4	20.7 ± 0.3	20.5 ± 0.2^d^
**% Body fat**	24.3 ± 0,7	22.10 ± 0.2	23,7 ± 0.8	22.7 ± 0.6	23.3 ± 0.4^d^
**Fat mass (Kg)**	12.1 ± 0.6	12.0 ± 0.5	11.8 ± 0.7	12.3 ± 0.5	12.2 ± 0.4^d^
**Fat-free mass (Kg)**	35.50 ± 0,5	39.0 ± 0.5	34.9 ± 0.6	39.7 ± 0.5^b^	37.6 ± 0.4
**AD-SoS**	2,012 ± 5.0	1,959 ± 5.1^a^	1,974 ± 6.0	1,997 ± 5.2^c^	1,988 ± 3.4
**UBPI**	0.73 ± 0.13	0.62 ± 0.12^a^	0.64 ± 0.01	0.71 ± 0.01^b^	0.69 ± 0.009^d^
**Flexibility (cm)**	22.7 ± 0.6	19.8 ± 0.6^a^	21.4 ± 0.8	21.1 ± 0.6	21.3 ± 0.4
**Abdominal (n)**	25.4 ± 0.8	33.5 ± 0.7	28.0 ± 0.9	30.1 ± 0.7	29.4 ± 0.6^d^
**Horizontal jump (cm)**	126.5 ± 1.6	147.4 ± 1.5	135.7 ± 1.9	138.2 ± 1.5	136.5 ± 1.3^d^
**VO**_**2 peak**_**(ml/Kg/min)**	40.7 ± 0.3	42.9 ± 0.3	41.6 ± 0.4	42.0 ± 0.3	41.8 ± 0.2^d^

Results are given as the mean ± SD.

^a^differences between sex (p < 0,01); ^b^ differences between pubertal stages (< 0,01); ^c^ differences between pubertal stages (< 0,05); ^d^ interaction sex × pubertal stage.

### Correlation between AD-SoS or UBPI and independent variables

**Figure 1:**
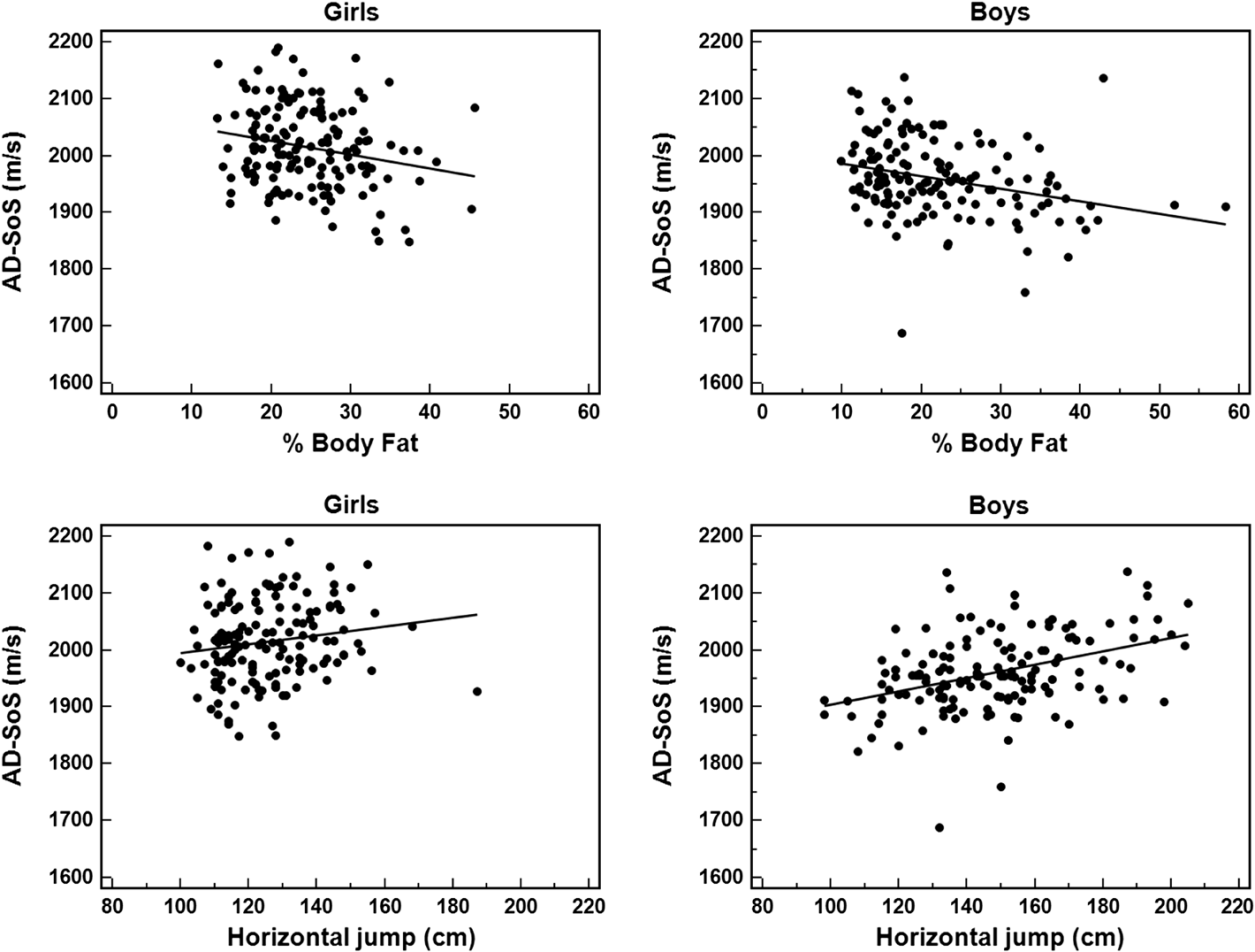
Correlation between percent body fat and the horizontal jump test with AD-SoS in both sexes.

Correlations between QUS parameters with anthropometric, body composition, and physical fitness variables are shown in Table 2. In both sexes, weight and height showed moderate positive correlations with AD-SoS and UBPI. In girls, %BF showed moderate negative correlations with AD-SoS and UBPI, but BMI only with AD-SoS as the horizontal jump was positively correlated with AD-SoS (Figure 1). For boys, AD-SoS and UBPI showed moderate positive correlations with fat-free mass.

**Table 2:** Pearson’s linear correlation coefficient of AD-SoS and UBPI according to sex, anthropometric variables, body composition, and motor and functional variables of 300 students

	**AD-SoS**				**UBPI**			
	**Female**		**Male**		**Female**		**Male**	
	**r**	**p**	**r**	**p**	**r**	**p**	**r**	**P**
**Age**	**0.522**	**0.002**	**0.330**	**0.000**	**0.270**	**0.000**	**0.399**	**0.000**
**Weight**	0.051	0.533	0.152	0.065	0.042	0.606	**0.162**	**0.049**
**Height**	**0.401**	**0.000**	**0.373**	**0.000**	**0.240**	**0.003**	**0.402**	**0.000**
**BMI**	**−0.172**	**0.034**	−0.059	0.475	0.070	0.394	−0.046	0.578
**% Body fat**	**−0.211**	**0.009**	**−0.317**	**0.000**	−0.125	0.125	**−0.274**	**0.001**
**Fat mass**	−0.092	0.262	−0.125	0.129	0.045	0.584	−0.089	0.280
**Fat-free mass**	**0.187**	**0.021**	**0.345**	**0.000**	0.121	0.137	**0.343**	**0.000**
**Flexibility**	0.072	0.377	0.048	0.563	0.117	0.151	−0.099	0.232
**Abdominal**	0.046	0.570	**0.315**	**0.000**	0.065	0.425	**0.341**	**0.000**
**Horizontal jump**	**0.166**	**0.041**	**0.406**	**0.000**	0.081	0.319	**0.309**	**0.000**
**VO**_**2 peak**_	−0.113	0.166	0.157	0.056	0.004	0.966	0.038	0.645

### Multiple regression analysis

Significant variables identified by the univariate analysis were further assessed in a stepwise multiple regression model. Separate models were developed for AD-SoS and UBPI and are shown in Table 3. In the model for the whole sample, sex, %BF, and height were identified as significant independent variables for AD-SoS and UBPI, age only for AD-SoS (adjusted r^2^ = 0.40; p < 0.001), and pubertal maturation for UBPI (adjusted r^2^ = 0.268; p < 0.001). In the male model, %BF and height were identified as significant independent variables for AD-SOS and UBPI, horizontal jump only for AD-SoS (adjusted r^2^ = 0.274; p < 0.001), and pubertal maturation only for UBPI (adjusted r^2^ = 0.295; p < 0.001). For girls, age and %BF were identified as significant independent variables for AD-SOS and UBPI (adjusted r^2^ = 0.093; p < 0.001), but height only for AD-SOS (adjusted r^2^ = 0.408; p < 0.001).

**Table 3:** Model of STEPWISE regression analysis with Ad-SoS and UBPI as dependent variables for male and female subjects

**AD-SoS**							**UBPI**						
**General**							**General**						
	**B**	**EP**	**B**	**R**^**2**^_**(adjusted)**_	**F**	**P**		**B**	**EP**	**β**	**R**^**2**^_**(adjusted)**_	**F**	**P**
Constant	1.081	0.140		0.400	7.709	0.000	Constant	0.897	0.155		0.268	5.799	0.000
Sex	−0.726	0.089	−0.365		−8.154	0.000	Sex	0.193	0.071	0,173		2.706	0.007
Age	0.270	0.065	0.242		4.172	0.000	Pubertal stages	−0.602	0.098	−0,303		−6.134	0.000
% Body fat	−0.320	0.046	−0.318		−6.888	0.000	% Body fat	−0.260	0.051	−0,260		−5.088	0.000
Height	0.280	0.060	0.278		4.693	0.000	Height	0.259	0.066	0258		3.939	0.000
**Male**							**Male**						
	**B**	**EP**	**β**	**R**^**2**^_**(adjusted)**_	**F**	**P**		**B**	**EP**	**β**	**R**^**2**^_**(adjusted)**_	**F**	**P**
Constant	−0.368	0.064		0.274	−5.764	0.000	Constant	−1.705	0.447		0.295	−3.812	0.000
Horizontal jump	0.169	0.078	0.184		2.168	0.032	Pubertal stages	0.555	0.175	0.273		3.168	0.002
% Body fat	−0.317	0.070	−0.345		4.508	0.000	% Body fat	−0.286	0.088	−0.278		3.262	0.001
Height	0.249	0.075	0.271		−3.331	0.001	Height	0.256	0.075	0.250		−3.436	0.001
**Female**							**Female**						
	**B**	**EP**	**β**	**R**^**2**^_**(adjusted)**_	**F**	**P**		**B**	**EP**	**β**	**R**^**2**^_**(adjusted)**_	**F**	**P**
Constant	0.353	0.059		0.408	5.992	0.000	Constant	−2.308	0.677		0.093	−3.409	0.001
Age	0.450	0.080	0.427		5.646	0.000	Age (years)	0.205	0.053	0.304		3.867	0.000
% Body fat	−0.365	0.063	−0.384		−5.769	0.000	% Body fat	−0.157	0.069	−0.178		−2.261	0.025
Height	0.276	0.075	0.290		3.674	0.000							

## Discussion

Our study provides specific QUS parameters that evaluate bone status (AD-SoS and UBPI) by sex and pubertal maturation and their association with physical fitness and body composition in Brazilian children and adolescents. It is the first research using phalange US and physical fitness in Brazilian adolescents.

Girls showed significantly higher QUS values than boys. Studies using DXA have shown that girls have higher BMD values than boys at trabecular sites [8,21,22]. The US method measures the distal portion of the proximal phalanges, which are rich in trabecular bone [23]. Regarding the pubertal stage, the pubertal group showed higher values than the prepubertal and intrapubertal groups. Ribeiro et al. [24] evaluated 1356 black and white students aged 6–11 years and found significant differences between the prepubertal and pubertal groups for AD-SoS and UBPI. These results were expected with respect to age, sex, and pubertal stage. They show that girls who experience early puberty have more bone mass than do same-age boys. Thus, sex hormones are important modulators of bone mass [25], suggesting an effect of estrogen on trabecular bone [26,27].

Several studies have investigated the association between the level of physical activity and bone mass in children and adolescents [5,28-31]. However, few studies have investigated bone mass and its relation to physical fitness. Our study showed that the variables related to muscle strength (fat-free mass, abdominal strength, horizontal jumping) contributed positively to the QUS parameters, whereas BMI and %BF contributed negatively (Table 2). Hence, the level of muscle strength appears to influence the AD-SoS and UBPI, confirming the hypothesis that an increasing level of fitness improves bone quantity [4,5] and quality, mainly in boys.

This effect on bone parameters related to physical strength variables is in accord with other studies. For instance, Ginty et al. [32] showed a positive association between the states of total and site-specific bone mineral, cardiorespiratory fitness, and muscle strength in male adolescents. Vicente-Rodriguez et al. [33] investigated the association between BMD with the physical fitness of 68 boys and girls. They found a direct association with cardiorespiratory fitness, muscular speed, and agility, suggesting that these results could have been due to the association of physical fitness and lean body mass. However, our study did not find a significant association between VO_2peak_ and QUS parameters. In fact, the results suggested that the bone mass differences between males and females could probably be explained by differences in physical fitness and lean mass [34,35]. The data in the literature regarding the association between physical fitness and bone status are still controversial. Although longitudinal studies have shown an increase in bone formation and reabsorption in adolescents caused by improved cardiorespiratory fitness [33,36], other studies showed that during adolescence and youth only neuromotor (muscular) fitness [31,37] was associated with BMD despite finding a significant correlation with cardiorespiratory fitness [38].

Regarding the variable fat, our study showed a negative correlation of AD-SoS and UBPI for %BF and BMI in boys and %BF for AD-SoS in girls. These results are consistent with those of other studies [38,39], which also found a negative correlation of fat to bone. The physiological basis to explain the relation between weight, body fat distribution, and bone mass remains unclear, particularly when considering different racial groups [22]. The results showing the adverse effect of increased fat mass on bone mass, along with significant positive associations of lean mass, corroborate the mechanostat theory described by some authors in which the geometry of the bone is adapted primarily by dynamic load imposed by muscle force—not to static loads represented by body weight [40].

The present study demonstrated that in a general regression analysis, sex and measurements related to growth (age, pubertal maturation, and height) were positive predictors for both AD-SoS and UBPI. In boys, the standing long jump and height were positive predictors for AD-SOS and pubertal maturation and height for UBPI. Conversely, for girls, age was a positive predictor for AD-SoS and UBPI but height only for Ad-SOS. An interesting finding was that for all groups the %BF was a negative predictor for both AD-SoS and UBPI. These findings are in accord with data from previous studies [22,24,40-42] and show that the body composition related to fat exerts a negative influence on bone mass in both sexes.

In our study, despite the lean mass having shown a relation with AD-SoS in boys and girls and with UBPI in boys, it did not appear as a predictor in the regression analysis. However, the horizontal jump as a positive predictor in boys showed that muscle strength positively influences AD-SoS, and the %BF has a negative influence on QUS parameters in both sexes.

The present study has some limitations. They include the large number of subjects lost from the original sample, self-evaluation of sexual maturation, no comparison of US data with DXA data, no implementation survey of fractures, and no evaluation of the ethnicity of the subjects.

## Conclusions

The results of the present study demonstrated that QUS of phalanges parameters are correlated with growth variables such as age, height, and pubertal maturation. Regarding physical fitness, only the variables related to muscle strength, especially in boys, showed an association with QUS parameters. In addition, fat mass demonstrated a negative association with AD-SoS and UBPI.

## Competing interests

The authors declare that they have no competing interests.

## Authors’ contributions

All of the authors have made substantial contributions to the study. AMM: conception and design of the study, acquisition of data, analysis and interpretation of data; drafting the article; final approval of the version to be submitted. EMG: acquisition of data, analysis and interpretation of data; revising the manuscript critically for important intellectual content; final approval of the version to be submitted. VJOB: interpretation of data; revising the manuscript critically for important intellectual content; final approval of the version to be submitted. GG-J: conception and design of the study; acquisition of data analysis and interpretation of data; revising the manuscript critically for important intellectual content; final approval of the version to be submitted. All authors read and approved the final manuscript.

## Pre-publication history

The pre-publication history for this paper can be accessed here:

http://www.biomedcentral.com/1471-2431/13/117/prepub
